# Editorial Notes, News and Miscellany

**Published:** 1913-02

**Authors:** 


### Editorial Notes, News and Miscellany.

BOOKS REVIEWED in the "Red Back" can be ordered at publishers' prices from or through this office; cash with order.

---

DRS. BENNETT, SCOTT & BIBB, Austin, Texas, have added to their splendid office equipment a Scheidel-Western "Interrupterless" transformer for X-ray work.

---

OUR VASOTOMY BILL is now before both houses of the Legislature. Dr. Daniel Parker, joint author of the bill, has charge of it in the House, and Senator Astin in the Senate. It is designed to prevent the breeding of the defectives whose progeny has nearly swamped the State, and we go on building palaces for them until the lunatics, in Texas alone, cost the taxpayers a million a year. I urge my readers to write to their representatives to support the bill. Eight or ten other States have already enacted similar laws.

P. S.—Since this was written, the bill has been reported favorably by the committees in both houses, and the prospect for passage is good. In a test case in September, 1912, the Supreme Court of the United States sustained a similar law and pronounced it constitutional. See Department of Eugenics, this issue.

---

DIABETES MELLITUS.—I am undertaking an exhaustive research into the pathology, etiology and dieto-therapy of diabetes mellitus. I am very anxious to hear from every physician in the United States who has a case under treatment, or who has had any experience in the treatment of this malady. Van Noorden says "the best treatment for the diabetic is the *food* containing the *greatest* amount of *starch* which the patient can bear without *harm*." If any physician who reads this has similar or contrary experience, and would take the trouble to write me, I would esteem it a special privilege to hear from him, if only a postal card.

Kindly address,

WILLIAM E. FITCH, M. D.,
355 W. 145 St., New York City.

